# Clonal Hematopoiesis of Indeterminate Potential and Cardiovascular Risk in Patients with Chronic Kidney Disease without Previous Cardiac Pathology

**DOI:** 10.3390/life13091801

**Published:** 2023-08-24

**Authors:** Maria Kislikova, Maria Ana Batlle Lopez, Francisco Javier Freire Salinas, José Antonio Parra Blanco, Maria Pilar García-Berbel Molina, Alejandro Aguilera Fernandez, Vicente Celestino Piñera Haces, Maria Teresa García Unzueta, Adalberto Benito Hernández, Juan Carlos Ruiz San Millan, Emilio Rodrigo Calabia

**Affiliations:** 1 Immunopathology Group, Nephrology Department, Marqués de Valdecilla University Hospital—IDIVAL, 39009 Santander, Spain; alejandroaguilerafdez@gmail.com (A.A.F.); vicentecelestino.pinera@scsalud.es (V.C.P.H.); adalberto.benito@scsalud.es (A.B.H.); juancarlos.ruiz@scsalud.es (J.C.R.S.M.); emilio.rodrigo@scsalud.es (E.R.C.); 2 Hematology Department, Marqués de Valdecilla University Hospital—IDIVAL, 39009 Santander, Spain; mana.batlle@scsalud.es; 3 Pathology Department, Marqués de Valdecilla University Hospital—IDIVAL, 39009 Santander, Spain; franciscojavier.freire@scsalud.es (F.J.F.S.); mpgarcia@idival.org (M.P.G.-B.M.); 4 Radiology Department, Marqués de Valdecilla University Hospital—IDIVAL, 39009 Santander, Spain; joseantonio.parra@scsalud.es; 5 Clinical Laboratory Department, Marqués de Valdecilla University Hospital—IDIVAL, 39009 Santander, Spain; mteresa.garciau@scsalud.es

**Keywords:** coronary artery calcification, heart disease, chronic kidney disease, major adverse cardiovascular events, clonal hematopoiesis of indeterminate potential, cardiovascular risk

## Abstract

Clonal hematopoiesis of indeterminate potential (CHIP) is defined by the clonal expansion of hematopoietic stem cells carrying certain genes associated with an increased risk of hematological malignancies. Our study analyzes the influence of CHIP on the risk of heart disease and cardiovascular events in a population with chronic kidney disease (CKD). A total of 128 patients were prospectively followed up for 18 months to detect major cardiovascular events (MACE). To detect the presence of silent heart disease, troponin I, NT-Pro-BNP, and coronary calcification were measured. A massive sequencing was performed to detect CHIP. A total of 24.2% of the patients presented CHIP, including that which was only pathogenic. The most frequently affected gene was TET2 (21.1%). Using multivariate logistic regression analysis, the presence of CHIP was not related to coronary calcification (OR 0.387, 95% CI 0.142–1.058, *p* = 0.387), nor was it related to troponin I or NT-Pro-BNP. A total of nine patients developed major cardiovascular events. Patients with CHIP did not have a higher risk of major cardiovascular events, although patients with DNMT3A did have a higher risk (HR 6.637, 95% CI 1.443–30.533, *p* = 0.015), independent of other variables. We did not find that CHIP was associated with a greater risk of silent heart disease or cardiovascular events, although those affected by DNMT3a, analyzed independently, were associated with a greater number of cardiovascular events.

## 1. Introduction

Chronic kidney disease (CKD) is an inflammatory disease with a high prevalence in all populations of the world, affecting around 9% of the adult population [1]. Although medical attention and research on CKD have focused mainly on the disease in end-stage renal disease, a shift in the focus of care and research to improving cardiovascular outcomes is now recommended because the majority of CKD patients die due to heart disease [2]. Ischemic heart disease, sudden death, and heart failure are highly prevalent in patients with CKD and contribute to increasing mortality and morbidity. In addition, heart disease in patients with CKD shows a different behavior, which makes diagnosis and treatment difficult [3]. A lower GFR and higher albuminuria are related to a higher prevalence of these risk factors [3]. In addition, some epigenetic changes secondary to the uremic milieu have recently been associated with cardiovascular disease in patients with CKD [4,5].

Clonal hematopoiesis of indeterminate potential (CHIP) is defined by the clonal expansion of hematopoietic stem cells carrying mutations in certain genes that are independently associated with increased risks of hematologic malignancies, atherosclerotic cardiovascular disease, worse heart failure outcomes, and all-cause mortality [6]. It is known that the development of CHIP is related mainly to aging but also to chronic inflammation, which confers a survival advantage to hematopoietic cells with CHIP mutations [6,7,8]. In turn, these cells accelerate chronic inflammation, and this positive feedback increases the risk of cardiovascular diseases [7,8,9]. To date, few studies have established a relationship between the incidence of CHIP mutations and renal function and its evolution, with some researchers reporting that the presence of CHIP is related to worse glomerular filtration (GFR) and a faster progression of CKD, while other authors have not observed this relationship [10,11,12]. Furthermore, no studies have analyzed whether CHIP in patients with nephropathy contributes to increased cardiovascular risk.

Our hypothesis was that, because it is an inflammatory disease related to aging, CKD may favor the appearance of these mutations in the transcriptional regulatory genes that define CHIP. These mutations may contribute to the pathogenesis of CKD-associated heart disease or could be useful as new CKD biomarkers. To test this hypothesis, we conducted a prospective study in a cohort of CKD patients without previously known cardiovascular diseases, analyzing the presence of subclinical heart disease (coronary artery calcification, troponin, and NR-ProBNP) at the time of CHIP determination and the subsequent appearance of clinical heart disease.

## 2. Materials and Methods

Patients with chronic kidney disease being monitored in nephrology clinics (nephrology outpatient clinics; chronic kidney disease clinics; peritoneal dialysis; and hemodialysis, except for transplantation) were included between 1 September 2020 and 31 January 2021. They had to be older than 18 years old and interested in participating in the study, as well as willing to sign the informed consent. The exclusion criteria were previous cardiovascular pathology, atrial fibrillation, and previous diagnosis of any neoplasia (including hematological). In total, 128 patients gave their consent to participate in the study. The study was approved by the Cantabria Clinical Research Ethics Committee (2020:326).

Coronary artery calcification obtained via computed tomography using 64/128 Optima (64) and Revolution EVO (128) detectors, GE Healthcare, USA, was measured with the prospective acquisition technique with heart rate monitoring. The amount of calcium in the coronary arteries was measured using SmartScore^®^ software (General Electric Healthcare, USA), and the amount of calcium was expressed according to the system proposed by Agatston. Agatston’s classification is defined as follows: 0–100, 100–200, over 400, etc. To carry out the study, the total amount of coronary calcium and the amount observed in the anterior descending artery were evaluated.

Together with the collection of anthropometric and clinical data, a blood sample was taken the same day in a standardized manner, in which creatinine (estimated glomerular filtration rate using the CKD-EPI equation), calcium, phosphorus, PTH, and LDL cholesterol were immediately analyzed; HDL cholesterol was analyzed via standardized, automated analysis in an Atellica CH Solution equipment and an Atellica IM Solution equipment (Siemens^®^ Healthineers, Tarrytown, NY, USA); and vitamin D (25-OH-vitamin D) was analyzed via automated chemiluminescence in a Liaison XL^®^ (DiaSorin). In addition, a frozen serum sample was kept at −80 °C for specific determinations (Troponin I, NT-proBNP). Troponin I (High Sensitivity Troponin I) and NT-pro BNP were performed using automated immunoassay on an Atellica^®^ IM (Siemens^®^ Healthineers, Tarrytown, NY, USA).

To detect CHIP, a venous blood sample was obtained in EDTA tubes and processed via mild lysis following standard procedures for the removal of red blood cells, and subsequently, the DNA was isolated from leukocytes with the QIAcube DNA Midi Kit (Qiagen, Hilden, Germany) in QIAcube (Qiagen). DNA concentrations were obtained using the Qubit dsDNA HS Assay Kit (Life Technologies, Carlsbad, CA, USA) in a Qubit 4 Fluorimeter (Life Technologies). The massive sequencing study was performed with Ion Chef System On-Demand technology using the Ion AmpliSeqTM Kit for Chef DL8 (Ion Torrent Cat. No. A29024). The panel design was performed at AmpliSeq.com for the TET2, DNMT3A, and MPL genes, with a total number of 161 amplicons, with full coverage of the coding region +/- 10 intronic bases to cover splicing regions. The in silico coverage was 100%, although a real coverage of 97% was obtained. In the first step, gene libraries were prepared using 2 pools of primers, 20 amplification cycles, and an alignment time and an extension of 4 min. In the second step, sequencing via synthesis was carried out on a 520 chip (Ion Torrent Cat. No. A27762); 32 patients with minimum coverage of 1000× and an analytical sensitivity of 4% were analyzed on each chip. Torrent SuiteTM software was used for data analysis, and Varsome software was used to record pathogenicity, which was assessed according to the ACMG criteria [13]. 

Patients were prospectively followed up until 30 June 2022. A major cardiovascular event (MACE) was defined if the patients presented an acute myocardial infarction requiring PTCA/stent, coronary bypass, stroke, transient ischemic attack, peripheral arterial ischemia requiring PTCA/stent, amputations, limb bypass, arterial thrombosis, cardiogenic shock, or cardiac arrest.

Our main objective was to assess the relationship between the presence of mutations in the transcriptional regulatory genes that define CHIP and the occurrence of cardiovascular (CV) events. The secondary objective was to analyze the relationship between the presence of CHIP and the presence of subclinical heart disease assessed via imaging (coronary artery vascular calcification with Agatston ≥ 400) or laboratory tests (Troponin I and NT-proBNP determination).

Continuous variables were expressed as the mean and standard deviation. Categorical variables were described as relative frequencies. The characteristics of patients with and without CHIP and with and without a global coronary calcification value measured by an Agatston score of ≥400 were compared by comparison of means using a t-test for continuous variables and Chi-square for categorical variables. Variables independently related to an Agatston global coronary calcification score of ≥400 were analyzed using multivariate logistic regression. Variables related to the development of MACE were analyzed using Kaplan–Meier analysis and univariate and multivariate Cox regression. Statistical analysis was performed using SPSS version 15.0 (SPSS, Inc., Chicago, IL, USA).

## 3. Results

### 3.1. Description of the Sample and Clinical Events

The presence of CHIP was determined in 128 patients, with a mean age of 61 ± 7 years, and a coronary calcification study was performed on 127 due to the death of a participant before carrying out the radiological study. Table 1 shows the characteristics of the population studied and the prevalence of CHIP in 47 patients (36.7%) presenting CHIP, including pathogenic and probably pathogenic mutations (24.2% considering exclusively pathogenic mutations), with TET2 being the most frequently affected gene (21.1%). Most of the mutations are de novo observed mutations, and just one DNMT3A p.Arg882Cys has been published previously; all of them produce an alteration in function. Complete list of mutations can be seen in supplementary material. 

Table 1 shows the differences between patients with and without CHIP. The mean of the frequencies of the allelic variant of the most frequent mutation in each patient was 14 ± 9%. Of the patients with CKD not included in dialysis, 18.75% started renal replacement therapy. Of them, 8.34% received a pre-empty kidney transplant. Of the patients who underwent renal replacement therapy, 37.5% received a kidney transplant. During follow-up (median 19 months), nine patients developed MACE, and nine died.

### 3.2. CHIP’s Relationship with Coronary Calcification

The presence of CHIP was associated with a lower risk of global and anterior descending coronary calcification and was not associated with other variables related to silent heart disease, such as troponin and NT-ProBNP. The prevalence of CHIP was not higher in older patients with a more advanced degree of CKD or in patients with stage 5 CKD, and it was lower in diabetics. The relationship between the different variables and global coronary calcification, established as an Agatston value of ≥400, is described in Table 2. Using multivariate logistic regression analysis with the variables that were significant in the univariate study (CKD stage 5, diabetes mellitus, LDL-cholesterol, phosphorus, and CHIP), CHIP did not maintain the relationship with coronary calcification (OR 0.387, 95% CI 0.142–1.058, *p* = 0.064), while diabetes mellitus (OR 6.419, 95% CI 2.436–16.915, *p* < 0.001) and phosphorus (OR 1.696, 95% CI 1.137–2.529, *p* = 0.010) were independently associated with the risk of coronary calcification being ≥400 (Hosmer–Lemeshow test: χ2 = 6.071, degrees of freedom 8, *p* = 0.639). Performing the same analysis and substituting CHIP for the presence of TET2 mutations, this variable was also not independently related (OR 0.341, 95%CI 0.085–1.3718, *p* = 0.130).

### 3.3. CHIP’s Relationship with Cardiovascular Events

**Figure 1:**
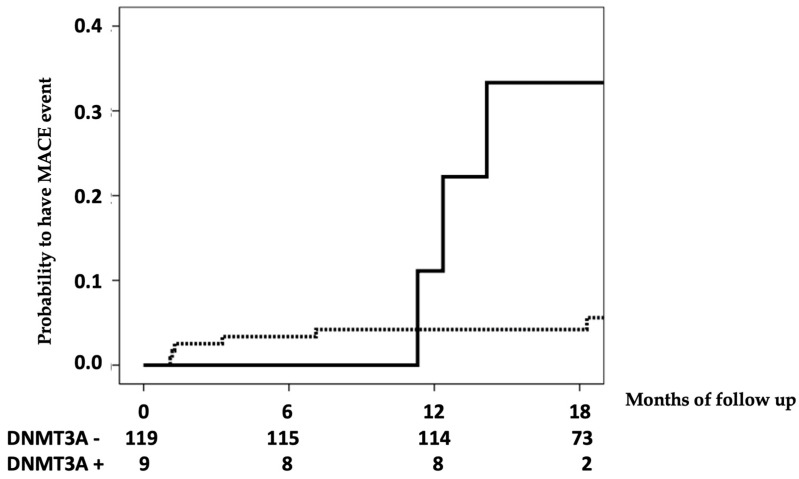
Development of cardiovascular events in patients with DNMT3A mutations (solid line) and without them (dashed line). In the table below the figure, the number at risk by time can be seen. Log-rank *p* = 0.001.

Table 3 shows the analysis of the variables related to the risk of MACE using univariate Cox regression. Although patients with CHIP did not have an increased risk of cardiovascular disease (CVD), patients with only DNMT3A mutations did have an increased risk of cardiovascular events (Figure 1); this risk was independent (HR 6.637, 95% CI 1.443–30.533, *p* = 0.015) of those with CHIP, coronary calcifications, stage 5 CKD, and phosphorus.

## 4. Discussion

In our group of patients with CKD without previous cardiovascular pathology, we did not find a relationship between the presence of CHIP and silent heart disease detected as coronary calcification or blood markers associated with heart disease, such as troponin I and NT-ProBNP. In fact, in the univariate study, CHIP seemed to protect against the risk of coronary calcification. This finding was due to the lower prevalence of diabetics in our group of patients with CHIP, demonstrating that the protective effect of CHIP (or individual gene mutations) on the risk of coronary calcification disappeared after the multivariate study. A previous study in the general population observed that there was indeed a relationship between the presence of CHIP and an increased risk of coronary calcification. Jaiswal et al. demonstrated that patients with CHIP without prior atherothrombotic disease in a Bioimage study had a more than threefold increased risk of having a coronary calcification value greater than or equal to 615, regardless of age, sex, the presence of diabetes or hypertension, total cholesterol, HDL, or smoking [9]. Although it cannot be stated from the results of our study, the discrepancy between the study by Jaiswal et al. and our findings could be due to the fact that the formation of coronary calcification in the general population is promoted by partially different factors than that in patients with CKD [9,14]. The magnitude of coronary calcification in patients with CKD is disproportionately high compared with the general population, which suggests the influence of specific renal mechanisms in its genesis. In our study, it can be concluded that, at least in a cross-sectional study, neither coronary calcification nor other markers of silent heart disease are pathogenetically related to CHIP; we cannot rule out that they influence the subsequent appearance of heart disease as part of MACE, as suggested by prospective follow-up.

The markers selected to detect silent heart disease have been related to the appearance of subsequent cardiovascular events both in the general population and in patients with CKD. The presence of coronary calcification measured with the Agatston scale is known to be related to overall mortality and cardiovascular events through prospective studies that have included thousands of patients [15,16], offering additional information on traditional risk factors to predict CV risk [17,18,19]. In the CKD group, both in stage 5 and previous ones, different authors have shown that coronary calcification doubles the risk of CV events and increases the risk of overall and cardiovascular mortality more than threefold, independently of other variables [20,21,22]. Similarly, both troponin I and NT-ProBNP have been associated with the risk of cardiovascular events and death in patients without known heart disease [23,24]. In our study, we did not observe a relationship between the presence of CHIP and an increased risk of presenting silent heart disease detected by the markers mentioned above.

In their initial study including 17,182 patients, Jaiswal et al. found that the presence of somatic mutations of genes associated with hematological malignancies in peripheral blood cells was associated with an increased risk of overall mortality (HR 1.4; 95% CI 1, 1–1.8), ischemic heart disease (HR 2.0; 95% CI 1.2–3.4), and ischemic stroke (HR 2.6; 95% CI 1.4–4.8), subsequently corroborating this relationship between CHIP and greater atherogenesis in animal models [9,25]. Unlike these studies carried out in the general population, in our patients with different degrees of CKD, we did not find a relationship between the presence of CHIP analyzed globally and the occurrence of major cardiovascular events. Other studies carried out on selected groups, such as patients with systemic lupus erythematosus, have also not observed a relationship between CHIP and increased cardiovascular risk [26]. On the contrary, in our study, it was detected that DNMT3A mutations increased the risk of CVD by more than six times. This association, moreover, was shown to be independent of other variables already recognized as strongly associated with MACE, such as coronary calcifications, CKD stage 5, and phosphorus [22,27]. On the contrary, the most frequent mutations in TET2 and MPL were unrelated to subsequent events. Our findings are different from those reported by Jaiswal et al., who found that each of the genes was separately associated with increased cardiovascular risk, although this effect was only observed by grouping the patients included in three previous studies, and this association was not demonstrated if the studies were analyzed in isolation [9]. The precise mechanisms by which CHIP may increase cardiovascular risk are unknown, although it is suspected that myeloid and monocyte cell clones carrying mutations are more proinflammatory and stimulate the progression of vascular lesions [28]. Fuster et al. developed a TET2-deficient mouse model with increased progression of interleukin-1 beta-mediated atherosclerosis, while Sano et al. found that interleukin-1 beta expression was not increased in monocyte-lineage cells in which DNMT3a was knocked out by CRISPR, although the expression of interleukin-6 mRNA was increased [29,30]. On the other hand, DNMT3a and TET2 may also promote increased cardiovascular risk by regulating lipid and carbohydrate metabolism [31]. In any case, no studies have been carried out on model animals deficient in TET2 or DNMT3a with chronic kidney disease in order to discern whether the mutations of each gene separately have a different effect on the atherogenic risk in this population. DNMT3a and TET2 are known to possess antagonistic enzymatic activities, and their expression predominates in different cells. DNMT3A encodes a methyltransferase that catalyzes DNA methylation, being a critical epigenetic regulator of gene expression, predominantly expressed in macrophages. On the contrary, TET2 encodes a transcriptional regulator that increases DNA demethylation, playing a greater role in the differentiation of myeloid cells [31,32]. A recent study showed that mutations in TET2 and DNMT3a are associated with increased and decreased methylation, respectively. The methylation is not global but is in specific genes for each mutation and can likewise detect differential methylation patterns for each CHIP mutation. For example, a decrease in methylation due to a mutation of DNMT3a in a specific gene was associated with an increase in the expression of NEAT1, which is related to inflammation and atherosclerosis, while the increase in methylation associated with mutations in TET2 was associated with a decrease in the expression of STAT6, which is associated with greater instability of the atherosclerotic plaque [33]. If the relationship of DNMT3a mutations with increased cardiovascular risk in CKD patients is confirmed in larger studies, it could suggest the existence of new specific mechanisms for the progression of the disease, atherosclerosis in patients with nephropathies, and new therapeutic alternatives.

In our study, we did not find that the prevalence of CHIP was related to worse renal function (either as a continuous variable or for each stage of CKD or in stage 5 patients) or with other variables that could condition more chronic inflammation or a worse environment that would justify changes in DNA, such as smoking or changes in phospho-calcium metabolism. A previous study observed that patients with CHIP were associated with a lower cystatin-estimated glomerular filtration rate but, as shown in our study, not with the creatinine-estimated glomerular filtration rate [10]. In the mouse models described by Jaiswal and Sano, the presence of variations in TET2 and DNMT3a was associated with greater glomerulosclerosis and greater renal fibrosis after exposure to angiotensin II [9,30]. Thus, CHIP could be associated with a greater progression of renal function deterioration. The results of two studies analyzing this effect obtained conflicting results. While Vlasschaert et al. associated the presence of CHIP with a more than doubled risk of a 50% decrease in the estimated glomerular filtration rate at 5 years in 172 patients, a study carried out on 358 patients with diabetic nephropathy did not observe a worse renal evolution in these patients [11,12]. One of the subsequent objectives of our study will be to prospectively compare the evolution of renal function between patients with and without CHIP after 5 years of follow-up.

Our study has various limitations, the most important being the smaller sample size and the shorter follow-up time compared with other studies [9,10,11,12,25]. Even in the study by Jaiswal et al., the mutations of each of the genes analyzed separately were not significantly associated with increased cardiovascular risk until the pooled data from each of the three included substudies were analyzed [9]. Our study was a pilot study, and we are interested in increasing our sample size, follow-up time, and number of genes in the near future. In relation to the small sample size and low number of events, our analysis could benefit from techniques and newer statistics we do not currently have access to. In addition, the study design did not allow us to detect whether sustained exposure to an environment of chronic renal dysfunction favors an increase in the prevalence of CHIP. To determine whether CKD favors the appearance of CHIP, a study should be carried out with serial blood samples to detect CHIP as the deterioration of renal function progresses in each patient. Lastly, and as previously stated at the beginning of the discussion, the lower prevalence of diabetics in our group of patients with CHIP may interfere with the analysis of the relationship between CHIP and coronary artery calcification. Regarding strengths, we carried out two studies, one cross-sectional and the other prospective, to analyze the influence of CHIP. Importantly, the determination of CHIP was carried out at our hospital by the hematology and molecular pathology teams, who are experts in its determination, as it is incorporated into the routine of hematology patients.

## 5. Conclusions

In conclusion, in our CKD population without previous cardiovascular pathology, we did not find a relationship between silent cardiac pathology and the prevalence of CHIP mutations. However, we selectively found that mutations in one of the CHIP genes, DNMT3A, were associated with a high risk of major cardiovascular events independently of other well-established variables, while the rest of the genes were not. This differential effect of DNMT3A on the cardiovascular risk of CKD patients and the influence of renal dysfunction on the appearance of CHIP should be studied in future studies.

## Figures and Tables

**Table 1:** Characteristics of the study population.

	Total	No CHIP (*n* = 81)	CHIP (*n* = 47)	*p*
Age (years)	61 ± 7	61 ± 7	61 ± 7	0.949
Sex—male	54.7%	53.8%	55.3%	0.874
BMI (kg/m^2^)	28.3 ± 5.3	28.5 ± 5.5	28.0 ± 5.1	0.586
CKD stage				0.565
1	12.5%	9.9%	17.0%	
2	13.3%	12.3%	14.9%	
3	21.9%	21.0%	23.4%	
4	21.1%	25.9%	12.8%	
5	31.3%	30.9%	31.9%	
GFR (mL/min/1.73 m^2^)	38.1 ± 30.4	35.1 ± 29.2	43.2 ± 32.1	0.145
Etiology of CKD				0.425
Glomerular	22.7%	21.0%	25.5%	
Diabetic	17.2%	18.5%	14.9%	
Vascular	14.8%	13.6%	17.0%	
Hereditary	9.4%	9.9%	8.5%	
Interstitial	8.6%	7.4%	10.6%	
Systemic	6.3%	3.7%	10.6%	
Unknown	21.0%	25.9%	12.8%	
Diabetes mellitus	36.7%	43.2%	25.5%	0.045
Nonsmoker	31.3%	32.1%	29.8%	0.786
Troponin (ng/L)	15.9 ± 26.1	18.3 ± 31.2	11.7 ± 13.0	0.098
NT-ProBNP (pg/mL)	2306.8 ± 6340.2	2793 ± 7245	1468 ± 2306	0.197
LDL cholesterol (mg/dl)	93.7 ± 38.6	92.5 ± 35.2	95.8 ± 44.2	0.645
HDL cholesterol (mg/dl)	48.3 ± 16.4	45.9 ± 12.3	52.5 ± 21.2	0.057
Lipoprotein A (mg/dl)	40.2 ± 50.3	43.0 ± 54.1	35.6 ± 43.3	0.427
Calcium (mg/dl)	9.1 ± 0.7	9.0 ± 0.7	9.1 ± 0.6	0.442
Phosphate (mg/dl)	4.1 ± 1.3	4.1 ± 1.3	4.0 ± 1.3	0.603
PTH (pg/mL)	216.6 ± 255.7	238.3 ± 275.0	179.3 ± 216.0	0.210
Vitamin D (ng/mL)	20.8 ± 10.3	19.7 ± 10.4	22.7 ± 10.0	0.120
Calcification of anterior descendent artery *	222.7 ± 382.8	270.8 ± 427.7	140.6 ± 276.2	0.039
Global coronary calcification *	543.6 ± 1250.6	695.3 ± 1451.4	285.2 ± 637.8	0.033
Agatston ≥ 400 *	30.7%	38.8%	17.0%	0.010
MACE	7%	7.4%	6.4%	0.827
Global mortality	7%	8.6%	4.3%	0.349
CV mortality	2.3%	3.7%	0.0%	0.182
CHIP	36.7%	-	-	-
One gene mutation		-	-	-
TET2	21.1%			
DNMT3A	7.0%			
ASXL1	0.8%			
JAK2	1.6%			
MPL	12.5%			
Two gene mutations	6.3%	-	-	-

* 127 patients.

**Table 2:** Variables related to significant global coronary calcification (Agatston ≥ 400).

	Agatston < 400 (*n* = 88)	Agatston ≥ 400 (*n* = 39)	*p*
Age (years)	60 ± 7	62 ± 7	0.071
Sex—male	64.1%	50.0%	0.455
BMI (kg/m^2^)	28.0 ± 5.4	29.0 ± 5.3	0.351
CKD stage 5	25.0%	43.6%	0.036
GFR (mL/min/1.73 m^2^)	42.8 ± 31.1	28.2 ± 26.4	0.008
Diabetes mellitus	23.9%	66.7%	<0.001
Smoker	27.3%	41.0%	0.124
Troponin (ng/l)	12.0 ± 19.3	25.0 ± 36.1	0.040
NT-ProBNP (pg/mL)	1049.7 ± 2110.6	5157.2 ± 10,589.7	0.021
LDL cholesterol (mg/dl)	99.3 ± 41.4	81.5 ± 28.7	0.006
HDL cholesterol (mg/dl)	50.0 ± 17.4	44.6 ± 13.3	0.060
Lipoprotein A (mg/dl)	40.1 ± 53.6	41.1 ± 43.3	0.913
Calcium (mg/dl)	9.1 ± 0.7	9.1 ± 0.7	0.755
Phosphate (mg/dl)	3.8 ± 1.0	4.7 ± 1.7	0.004
PTH (pg/mL)	187.1 ± 187	258.6 ± 332.5	0.125
Vitamin D (ng/mL)	21.8 ± 9.4	18.9 ± 12.1	0.144
CHIP	44.3%	20.5%	0.010
Gene mutation			
TET2	27.3%	7.7%	0.013
DNMT3A	6.8%	7.7%	0.859
MPL	15.9%	5.1%	0.091

**Table 3:** Analysis of the variables related to the appearance of MACE using univariate Cox regression.

	HR	95%IC	*p*
Age (years)	1.051	0.950–1.163	0.335
Sex—male	0.216	0.045–1.043	0.056
BMI (kg/m^2^)	1.075	0.949–1.217	0.254
CKD stage 5	8.469	1.756–40.834	0.008
GFR (mL/min/1.73 m^2^)	0.916	0.842–0.997	0.043
Diabetes mellitus	3.532	0.882–14.143	0.075
Smoker	2.796	0.750–10.415	0.125
Troponin (ng/l)	1.000	0.977–1.024	0.977
NT-ProBNP (pg/mL)	1.090	1.050–1.132	<0.001
LDL cholesterol (mg/dl)	0.989	0.969–1.010	0.311
HDL cholesterol (mg/dl)	0.908	0.836–0.987	0.024
Lipoprotein A (mg/dl)	1.002	0.991–1.014	0.717
Calcium (mg/dl)	0.451	0.194–1.048	0.064
Phosphate (mg/dl)	1.741	1.284–2.361	<0.001
PTH (pg/mL)	1.002	1.000–1.003	0.014
Vitamin D (ng/mL)	0.961	0.893–1.034	0.290
Calcification of anterior descendent artery *	1.002	1.001–1.003	<0.001
Global coronary calcification *	1.001	1.001–1.001	<0.001
Agatston ≥ 400 *	8.223	1.012–66.842	0.049
CHIP	0.848	0.212–3.393	0.816
Gene mutation			
TET2	0.034	0.000–30.509	0.330
DNMT3A	7.343	1.817–29.673	0.005
MPL	0.040	0.000–202.888	0.460

* 127 patients.

## Data Availability

The data presented in this study are available on request from the corresponding author. The data are not publicly available due to publishing pilot study data.

## Supplementary Materials

The following supporting information can be downloaded at: https://www.mdpi.com/article/10.3390/life13091801/s1, Table S1: List of CHIP detected mutations.
